# The Relationship between Psychological Well-Being and Psychosocial Factors in University Students

**DOI:** 10.3390/ijerph17134778

**Published:** 2020-07-02

**Authors:** Francisco Manuel Morales-Rodríguez, Isabel Espigares-López, Ted Brown, José Manuel Pérez-Mármol

**Affiliations:** 1 Department of Educational and Developmental Psychology, Faculty of Psychology, Campus Universitario de Cartuja, University of Granada, 18071 Granada, Spain; fmmorales@ugr.es; 2 Department of Legal Medicine, Toxicology and Psychiatry, Faculty of Medicine, Avda. de la Investigación, 11, 18016 Granada, Spain; isaespigares@correo.ugr.es; 3 Department of Occupational Therapy, Faculty of Medicine, Nursing and Health Sciences, Monash University—Peninsula Campus, Frankston, VIC 3199, Australia; ted.brown@monash.edu; 4 Department of Physiotherapy, Faculty of Health Sciences, University of Granada, Avda. de la Ilustración, 60, 18016 Granada, Spain; 5 Instituto de Investigación Biosanitaria de Granada (ibs.Granada), 18016 Granada, Spain

**Keywords:** psychological well-being, university students, learning styles, social skills, emotional intelligence, anxiety, empathy, self-concept

## Abstract

Determining what factors influence the psychological well-being of undergraduate university students may provide valuable information to inform the development of intervention programs and targeted learning activities. The objective of this study was to investigate the correlation between psychological well-being in university students and their self-reported learning styles and methodologies, social skills, emotional intelligence, anxiety, empathy and self-concept. The final sample consisted of 149 Spanish university students, with an average age of 21.59 years (*SD* = 4.64). Psychological well-being dimensions, along with learning style and methodology preferences, social skills, level of social responsibility, emotional intelligence, state and trait anxiety, empathy and levels of self-concept were measured using a series of validated self-report scales. The results indicate that the total variance explained by the university students’ psychological well-being factors were as follows: i) self-acceptance dimension (R^2^ = 0.586, *F*(6,99) = 23.335, *p* < 0.001); ii) positive relationships dimension (R^2^ = 0.520, *F*(6,99) = 17.874, *p* < 0.001); iii) autonomy dimension (R^2^ = 0.313, *F*(4,101) = 11.525, *p* < 0.001); iv) environmental mastery dimension (R^2^ = 0.489, *F*(4,101) = 24.139, *p* < 0.001); v) personal growth dimension (R^2^ = 0.354, *F*(4,101) = 13.838, *p* < 0.001); and vi) purpose-in-life dimension (R^2^ = 0.439, *F*(4,101) = 19.786, *p* < 0.001). The study findings may be used to inform new educational policies and interventions aimed at improving the psychological well-being of university students in the international context.

## 1. Introduction

### 1.1. Psychological Well-Being

Psychological well-being has been defined within the eudaimonic perspective as the development of one’s true potential. This is in contrast to the subjective well-being view [1,2]. Psychological well-being is viewed as the result of a life well-lived and is an important factor in students successfully adapting to college/university life. For this reason, this construct usually includes dimensions such as self-acceptance, positive relationships, autonomy, environmental mastery, personal growth and purpose-in-life [3]. Students attending university have to adjust to a new learning context and are under increased academic pressure [4,5]. This stage is considered to be one of the highest anxiety and lowest psychological well-being phases in the life cycle, with high levels of psychological distress compared with the general population [4,6]. Several studies have reported lower levels of psychological well-being in students attending university [3,7,8]. In a recent study by Sandoval et al. [8], a high percentage of university students exhibited a medium degree of psychological well-being, indicating that it is an indicator of their degree of adjustment adaptation.

### 1.2. Psychosocial Factors

Several models support the possible psychosocial factors related to psychological well-being. From a general perspective, the psycho-educational approach is an integral framework for the development and evaluation of psychological and educational constructs such as social skills, empathy, self-concept, anxiety and emotional intelligence, among others [9,10,11]. More specific frameworks, such as EuroPsy for the development of standards for high-quality professional education in psychology, include the following higher education competencies: adequate levels of empathy or anxiety, socially responsible attitudes, emotional management, problem solving and learning style preferences [11]. Other frameworks, such as the European Higher Education Area [10] and the Organization for Economic Cooperation and Development [9], highlight the importance of developing systemic competencies that include inter- and intra-personal psychological resources such as emotional intelligence, self-esteem, social skills, social responsibility and empathy [9,10]. From a psychological perspective, emotions are key constructs related to psychological well-being and satisfaction with life, according to classical theoretical models such as that of Goleman [12], Mayer et al. [13] and Bar-On [14]. Other more contemporary models include those by Bisquerra and Pérez-Escoda [12,15,16,17,18]. These models propose that emotional/psychological skills may be divided into two poles inside a continuum. The positive side includes elements such as self-esteem, social skills and empathic attitudes, and the negative side includes symptoms such as anxiety [5,19,20,21,22]. Both sides seem to have a relationship with psychological well-being, according to the existing empirical literature [5,19,20,21,22,23,24,25].

From an educational perspective, learning style preferences are understood as the various ways of overcoming, planning and resolving the demands of learning. There are four learning styles based on the preferences of the individual: the activist style based on direct experience, the reflector style based on observation and data collection, the theorist style based on abstract conceptualization and conclusion formation and the pragmatist style based on active experimentation and a search for practical applications [26]. In relation to learning methodologies, two types are usually differentiated, traditional learning, usually more common in education, whose methodology is expository, individualistic and competitive, and cooperative learning, which is characteristically more autonomous, social and dialectical. García-Ruiz and González Fernández [27] noted that the cooperative learning methodology was more positive for students than the traditional approach, since their learning was greater and of better quality. Cooperative learning methodologies usually influence the degree of academic satisfaction [5,28]. In turn, the greater the satisfaction with the academic environment in general (contents of university subjects, types of assessments used, methodologies applied to learning, teaching / learning styles, etc.), the larger the psychological well-being perceived by university students [22].

Under the umbrella of educational resources, social skills are the ability to adequately manage interpersonal relationships with the environment and to correctly understand, control and adjust interpersonal strategies. Social skills are measured by the overall social competence of the individual and by the inter- and intra-personal strategies used [29]. These skills appear to be related to academic performance in the university environment [30]. Positive social relationships have been shown to be associated with psychological well-being [31]. In addition, university social responsibility has been studied from the perspective of the organization, understanding that it should meet the expectations of stakeholders such as current and/or future students [32]. However, the importance of the individual perspective of the students’ values should be emphasized [33].

On the positive side of the psychological continuum, emotional intelligence interconnects emotions with reason, or in other words, emotions influence our thoughts, just as our cognitive processes influence our emotional states [34]. The concept of emotional intelligence is defined as the cognitive abilities that can be measured through tasks involving the processing of emotional information. This has been developed at a theoretical and empirical level, to demonstrate its predictive ability in different areas of daily life [35,36]. The latest research on emotional intelligence highlights its role in the ability of individuals to adapt to daily life environments and is linked to well-being [36,37,38,39]. In the tertiary context, the implication of high emotional intelligence in relation to academic performance in university students has been studied, emphasizing the role of emotional skills [24]. Some authors have highlighted the importance of emotional intelligence as a type of psychosocial adaptation in the university educational environment [38], it being a possible predictor of psychological well-being [23]. Several studies involving university students have examined the impact of proficient emotional intelligence skills in relation to academic performance, highlighting the key role of emotional skills [24]. This indicates the key role of emotional intelligence and its related dimensions (such as empathy) in university teaching and learning environments with students.

Empathy is the skill that allows us to know how other people are feeling, what they are thinking, understand their intentions, predict their behavior and understand their emotions [39]. Some studies on empathy have focused on analyzing it in young people, as it contributes to the enhancement of social skills and prosocial behavior [40,41]. The psychological well-being perceived by students appears to be strongly associated with empathy. Gustems Carnicer and Calderón [42] conducted a study with a group of university students where they found that students at high risk of psychological distress had higher scores for empathic stress and avoidance coping strategies. On the one hand, they obtained a direct correlation between psychological distress and emotional discharge, cognitive avoidance, the search for alternative rewards and resignation [42]. Recent studies have reported relationships between the emotional ability known as empathy and subjective well-being in university students [5,43,44]. Self-concept is considered a complex term because of the difficulty of differentiating it from similar terms that have even been used as synonyms, such as self-esteem [45]. Several authors refer to this as the labels that people give themselves, generally related to their physique, behavior and emotions [46]. Behavioral, affective and social functioning are explained by the perception of an individual’s experiences; therefore, one’s self-concept could be a predictor of one’s psychological well-being [45,47]. In a study conducted with university students in the area of self-concept within a cooperative learning structure, there was an improvement in self-concept [48]. Other studies [49] have reported positive relationships between psychological well-being and physical self-concept and self-esteem [22,49,50,51]. Therefore, self-concept could be included in studies as a probable factor related to university students’ psychological profiles.

On the negative side of the psychological continuum, anxiety has been erroneously considered synonymous with other concepts such as stress, fear or distress. Spielberger et al. [52] defined anxiety as an emotional reaction that is externalized through tension, apprehension, nervousness and worry, in addition to activation of the autonomous nervous system. Spielberger, Gorsuch and Lushene [53] establish two types of anxiety based on lengths of time: state and trait anxiety. For Spielberger [54], state anxiety refers to an immediate emotional state, modifiable over time, while trait anxiety is a relatively stable disposition, tendency or personality trait. Different concepts of anxiety (state and trait) need to be studied. According to Sandín and Chorot [55], anxiety implies at least three response systems (cognitive, physiological and behavioral), with their activation creating a fight or flight response, which in turn can have an effect at the psychological level [55]. The vast majority of the scientific literature suggests that approximately 50% of university students have experienced significant levels of anxiety [56,57]. Research focused on the university population concludes that the effects of anxiety are closely related to certain variables such as academic performance, abandoning the course and psychological and emotional well-being [58,59]. However, it has not been studied in conjunction with other explanatory variables of psychological well-being.

The study of the potential predictive relationship between the psychosocial factors previously described and psychological well-being in a university student sample provides a more holistic view for prospective educators, researchers and health care practitioners. Findings from this study may inform the development of new educational policies and intervention programs aimed at directly improving the psychological well-being of university students in the international context. Likewise, studies of this type could strengthen lines of research oriented towards the application of intervention programs aimed at the well-being of students and their academic performance. Conducting studies with sample groups of first-year undergraduate students from social and health areas allows a suite of baseline educational and psychosocial data measures to be collected on which intervention programs can be founded. This is beneficial in two key ways. Firstly, the objective data can be used to determine what psychosocial and educational factors need urgent attention and remediation activities put in place in courses such as social education, pedagogy or speech therapy. Secondly, follow-up data can be then collected after program planning, implementation and completion to determine its efficacy in those disciplines. The information about the possible potential relationship between psychological well-being and psychosocial factors provides a landscape overview about potential strategic changes that are needed in this higher education context over the duration of enrolment of first-year students up until they finish their degree. This evaluation is useful to improve the process of adjustment, socio-emotional adaptation to the university context and the quality of life of first-year students to better equip them with the foundation skills needed to be successful upon graduation and during the first few years of their work life. In sum, collecting baseline data from first-year students can have both short- and long-term benefits for the designer of intervention programs and for the students themselves.

### 1.3. Research Hypothesis

The research hypothesis of this study is as follows: psychological well-being dimensions are significantly related to a multifactorial construct composed of psycho-educational dimensions, such as educational aspects (learning style and methodology, social skills and level of social responsibility) and psychological or cognitive-affective skills (emotional intelligence, anxiety, empathy and levels of self-concept).

### 1.4. Aims

To investigate the possible association between psychological well-being and psychological factors among undergraduate university students.

## 2. Materials and Methods

### 2.1. Design

A cross-sectional approach using standardized self-report scales.

### 2.2. Participants

The initial sample consisted of 164 participants, of whom 32.3% were men and 67.7% women. The sample was taken from the first year of the undergraduate degree courses in Social Education, Pedagogy and Speech Therapy at the University of Granada, Spain. After applying the selection criteria for the present study, the final sample was 149 university students (15 students were excluded). The inclusion criteria were: a) being a full-time university student enrolled in the first year of his/her course; and b) providing informed consent to take part in the study. The exclusion criteria included: a) not completing each and every one of the questionnaires provided; b) not being a full-time student, having a recognized part-time student status and/or having requested a single assessment; and c) being a student with special educational needs. The present study protocol was approved by the Ethics Committee of the University of Granada (Granada, Spain), with the registration number: 328/CEIH/2017. The participants completed an individual informed consent form to participate in the study.

### 2.3. Instruments

Demographic data were collected through a self-report questionnaire. It included items asking questions about the following information: age, sex, academic program, year and semester of course. There was an evaluation of psychological well-being, learning styles, learning methodologies, social skills, social responsibility, emotional intelligence, anxiety, empathy and levels of self-concept. All the instruments used in the study were validated in Spanish language versions: they were available and were completed in Spanish.

#### 2.3.1. Psychological Well-Being

The starting point was Ryff’s Psychological Well-Being Scale [1,22]. The Spanish adaptation was provided by Díaz et al. [3], who assessed six dimensions of psychological well-being: self-acceptance: as the individual’s attempt to feel good about themselves; positive relationships: understood as the capacity to love, where social relationships are stable and trustworthy; autonomy: described as self-determination, independence and personal authority; environmental mastery: involving managing the demands and opportunities of the environment to satisfy one’s own needs and capacities; personal growth: where there is an effort to develop one’s capabilities and maximize them; and purpose in life: it consists of the need to set goals and define objectives to give life meaning. The scale consists of 39 items that use a Likert-type response format, ranging from one (total disagreement) to six (total agreement). A higher score means that the person shows higher levels of well-being. The Spanish version of the instrument has adequate reliability and validity properties for the six dimensions (RMSEA = 0.07). Internal consistency coefficients for the subscales of the Spanish version for the sample of this study were as follows: self-acceptance, 0.88; positive relationships with others, 0.72; autonomy, 0.90; environmental mastery, 0.89; purpose-in-life, 0.88; and personal growth, 0.94.

#### 2.3.2. Learning Styles and Methodologies

The Honey-Alonso Learning Styles Questionnaire [26] evaluates the psychological, affective and physiological characteristics expressed by a person when faced with a learning situation. Learning styles are divided into four dimensions: 1) active style, based on direct experience, 2) reflexive style, based on observation and data collection, 3) theoretic style, based on abstract conceptualization and conclusion formation and 4) pragmatic style, based on active experimentation and the search for practical applications. The questionnaire is composed of 80 items that use a dichotomous response format. The maximum score is 20 points for each style. The Spanish version of the instrument exhibited adequate validity properties for the 4 dimensions after factorial analysis. The active style explained 41% of total variance, the theoretic style 39.5%, the pragmatic style 40.2% and reflexive style 42.7% [26]. The reliability results obtained for the sample of this study for the styles were a Cronbach alpha of: 0.88 (active), 0.90 (reflexive), 0.88 (theoretic) and 0.76 (pragmatic). Further, also used was the questionnaire on cooperative learning and traditional learning methods [41]. This questionnaire assesses the respondent’s skill acquisition level, based on the respondents’ preference for the two learning approaches. This instrument has adequate psychometric properties of reliability and validity. The questionnaire consists of 34 items. Internal consistency coefficients of Cronbach’s alpha of 0.92 for traditional learning, and 0.89 for cooperative learning have been reported.

#### 2.3.3. Social Skills

The social skills scale consists of 20 items, each item representing one inter- or intra-personal social skill, using a Likert scale of five response options, ranging from one (never) to five (always) [29]. This instrument has adequate psychometric properties of reliability and validity. The study of the validity of this instrument yields satisfactory results. The reliability analysis for the present study showed a Cronbach alpha coefficient of 0.93. The social responsibility questionnaire [60] evaluates the self-attribution of socially responsible behavior. The questionnaire is divided into 40 items that use a Likert scale scoring format from one (never) to five (always). The higher the score, the more frequently an individual engages in socially responsible behavior. This instrument has adequate psychometric properties of reliability and validity. The validity was verified by the inter-judge agreement method and was satisfactory [60]. A Cronbach alpha of 0.96 was obtained for this questionnaire.

#### 2.3.4. Emotional Intelligence

The Trait Meta-Mood Scale-24 (TMMS-24) [61,62] emotional intelligence questionnaire was used. This questionnaire evaluates perceived emotional intelligence, understood as the ability to control feelings and emotions, to discriminate between them, and to use that ability to direct one’s own thoughts and actions. The instrument has three dimensions: emotional attention, understood as the capacity to feel and adequately express feelings; clarity, interpreted as an optimal understanding of one’s own emotional states; and repair, which alludes to the capacity for optimal control of emotional states. The questionnaire consists of 24 items rated on a Likert scale from one to five points. In terms of validity, all three factors were correlated appropriately and in the expected direction with classical criteria variables such as depression, anxiety, rumination and life satisfaction [61]. The instrument has shown a high reliability in its three dimensions: Cronbach alpha of 0.87 for emotional attention, 0.78 for clarity and 0.57 for repair.

#### 2.3.5. Anxiety

The State-Trait Anxiety Inventory [63] was used. This inventory has been designed to evaluate two aspects of anxiety: anxiety as a state, understood as a transient emotional condition; and anxiety as a trait, attributed to a relatively stable propensity for anxiety. The questionnaire consists of 40 items that are rated using a four-point Likert response scale which depends on intensity, ranging from zero (almost never/nothing) to three (almost always/much) [63]. The Spanish version of the instrument has adequate psychometric properties of reliability and validity. The Cronbach’s alpha coefficient for the state anxiety subscale was 0.93, while for the trait anxiety subscale it was 0.88.

#### 2.3.6. Empathy

The Test of Cognitive and Affective Empathy—TECA [39] was used. This test evaluates cognitive-affective skills related to the level of empathy. The test consists of four dimensions. Within the cognitive area are the dimensions of perspective taking (capacity for tolerance, communication and personal relationships) and emotional understanding (ability to recognize and understand emotional states, intentions and impressions of others). The affective area includes empathic stress (connection with other people’s negative emotional states) and empathic happiness (ability to share other people’s positive emotions) [39]. The test consists of 33 items that are rated on a Likert scale from one (totally disagree) to five (totally agree). High scores in each dimension indicate a higher level of empathy. The Spanish version of the instrument has adequate psychometric properties of reliability and validity [39]. Cronbach’s alpha resulted in 0.87 for the global TECA and the higher value for the four dimensions was a Cronbach alpha of 0.63.

#### 2.3.7. Self-Concept

The AF5 Multidimensional Self-Concept Scale [64] was used. This scale is based on a model that views self-concept as a multidimensional construct organized hierarchically from a general dimension. This questionnaire evaluates five dimensions: academic/professional, social, emotional, family and physical. This instrument consists of 30 items, scored on a scale from 1 to 99 points, where 1 corresponds to total disagreement and 99 to total agreement. The higher the score, the better the self-concept. The Spanish version of the instrument has adequate psychometric properties of reliability and validity. The factor analysis satisfactorily confirmed the five theoretical dimensions, explaining 51% of the total variance [64]. The reliability of the total scale in this study using Cronbach’s alpha test was 0.86 and this was 0.90 for academic/professional, 0.50 for social, 0.82 for emotional, 0.53 for family and 0.83 for physical.

### 2.4. Procedure

Regarding recruitment of the sample, participation was voluntary, i.e., the consecutive recruitment of participants was performed. The questionnaires were completed by the student participants at the end of a scheduled morning class during the second semester, under the supervision of a research assistant. Once the general procedures and objectives of the study had been explained, instructions were given to complete the questionnaires in hard copy version. The students provided written consent and the confidentiality of the data obtained was assured. The questionnaires were field-tested beforehand. The average time it took participants to complete the questionnaire was two hours, split between two sessions. In each session, the participants took at least one voluntary five-minute break. The anonymity of the participants was guaranteed since the hard copy included only a number, and their personal data were preserved in a different document linking this number and their personal data. Power relationships from an ethical perspective were not present. The data were manually entered into a database.

### 2.5. Data Analysis

The SPSS software (version 22.0, IBM Corp, Armonk, NY, USA) was used for the statistical analysis. A descriptive analysis was performed and the normal distribution of the variables was confirmed. Student t-test and univariate analysis of variance (ANOVA) were completed to investigate the relationship between psychological well-being and sex and level of course, respectively. Pearson’s correlation coefficient was used to determine the association between psychosocial variables and each of the psychological well-being dimensions. We used multiple linear regression analysis to investigate the predictive relationship between emotional well-being as the dependent variable, and age and the psychosocial variables (learning style and methodology preferences, social skills, level of social responsibility, emotional intelligence, anxiety, empathy and levels of self-concept) as the independent variables. The regression analysis was also executed in an independent manner when differences between academic disciplines were found for any of the psychological well-being dimensions. Normality of residuals, homogeneity of variance for residuals and linearity of data were examined before completing the regression model. The data met all the assumptions required to carry out the multiple linear regression analyses. Multi-collinearity was avoided by selecting a stepwise method in the regression model. A *p* < 0.05 was used as the significance level in the study.

### 2.6. Sample Size

In a previous study completed by Atienza [22], a significant bivariate correlation of 0.298 between psychological well-being (self-acceptance dimension) and emotional intelligence (repair dimension), measured with the Ryff scale (assessing well-being) and the TMMS-24 scale (evaluating repair), was used to calculate the sample size required to detect this size effect in the sample. This was carried out using the G*power software (version 3.1, Institut für Experimentelle Psychologie, Düsseldorf, Germany). This calculation demonstrated that a sample size of 140 university students was needed to provide a confidence interval of 95%, with a power of 95%, assuming a bilateral significance level (α) of 0.05. To be able to handle possible missing data, any participants dropping out or badly completed instruments, the recruited sample should be increased by around 5%. As a result, the final sample should include at least 147 participants.

## 3. Results

### 3.1. Description of the Sample

The total sample consisted of 149 students enrolled on the Social Education (*n* = 52), Pedagogy (*n* = 56) and Speech Therapy (*n* = 41) courses at the University of Granada, of whom 67.7% were women and 32.3% men, with an average age of 21.59 (*SD* = 4.64) years. One hundred percent of the sample was recruited from the first year of the three courses. The descriptive statistics of the independent and independent variables of the sample are reported in Table 1.

### 3.2. Confounding Variables for Psychological Well-Being

No statistically significant differences between sexes in relation to psychological well-being were found since there were no differences between male and female participants in terms of self-acceptance (*t* = 0.285, *p* = 0.776), positive relations with others (*t* = -0.971, *p* = 0.333), autonomy (*t* = 0.929, *p* = 0.354), environmental mastery (*t* = 0.067, *p* = 0.947), purpose-in-life (*t* = −1.344, *p* = 0.181) and personal growth (*t* = −0.955, *p* = 0.343). Among the three student course groups, statistically significant differences in the scores for the psychological well-being dimensions of environmental mastery (F(2,101) = 3.682, *p* = 0.028) and purpose-in-life (F(2,101) = 4.631, *p* = 0.011) (ANOVA) were found.

Specifically, the post-hoc analysis showed differences between the student participants enrolled on the Pedagogy and Speech Therapy academic courses for environmental mastery (*p* = 0.037; Pedagogy M ± SD = 12.90 ± 0.62; Speech Therapy M ± SD = 10.56 ± 0.68) and purpose-in-life (*p* = 0.011; Pedagogy M ± SD = 21.19 ± 4.33; Speech Therapy M ± SD = 18.17 ± 5.69).

### 3.3. The Correlation between the Psychological Well-Being Dimensions and Psychosocial Factors

Bivariate correlation analyses have shown direct and inverse relationships between the psychological well-being dimensions and the cooperative learning methodology, the empathy dimension of emotional understanding, social skills and all dimensions of the levels of self-concept, emotional intelligence and levels of anxiety in university students. These results are shown in Table 2.

The dependent variables (each dimension on the Ryff’s Psychological Well-Being Scale) exhibited a normal distribution. After testing the normality of the residuals in the regressions, it was confirmed that the observed residuals were normally distributed. The independence of data for all regressions performed for each dimension of well-being was confirmed. There was a linear relationship between the independent variables and the dependent variables. Homogeneity of the residuals’ variance was not violated and the data met the assumption of homoscedasticity.

### 3.4. Influence of Psychosocial Factors on Psychological Well-Being in University Students

Multivariate regression analysis showed that the cooperative learning methodology, the clarity dimension of emotional intelligence, state anxiety, the emotional understanding dimension of empathy and physical and family self-concept were significantly related to the dependent variable, predicting 58.6% of the total variance (R^2^ = 0.586, F(6,99) = 23.335, *p* < 0.001) of levels of self-acceptance in university students. The active learning style, emotional understanding and empathic stress, academic/professional self-concept, social self-concept and family self-concept were significantly related to the dependent variable, predicting 52% of the total variance (R^2^ = 0.520, F(6,99) = 17.874, *p* < 0.001) of levels of positive relationships in this sample. Social skills, the emotional attention and clarity dimensions of emotional intelligence and emotional self-concept were significantly related to the dependent variable, predicting 31% of the total variance (R^2^ = 0.313, F(4,101) = 11.525, *p* < 0.001) of levels of autonomy. Global emotional intelligence, state anxiety, emotional understanding and family self-concept were significantly related to the dependent variable, predicting 48.9% of the total variance (R^2^ = 0.489, F(4,101) = 24.139, *p* < 0.001) of levels of environmental mastery. The emotional attention dimension of emotional intelligence, state anxiety, the emotional understanding dimension of empathy and academic/professional self-concept were significantly related to the dependent variable, predicting 35.4% of the total variance (R^2^ = 0.354, F(4,101) = 13.838, *p* < 0.001) of levels of personal growth. Global emotional intelligence, state anxiety, empathic happiness and family self-concept were significantly related to the dependent variable, predicting 43.9% of the total variance (R^2^ = 0.439, F(4,101) = 19.786, *p* < 0.001) of levels of purpose-in-life. There was no collinearity between the variables included in the regression model.

**Figure 1:**
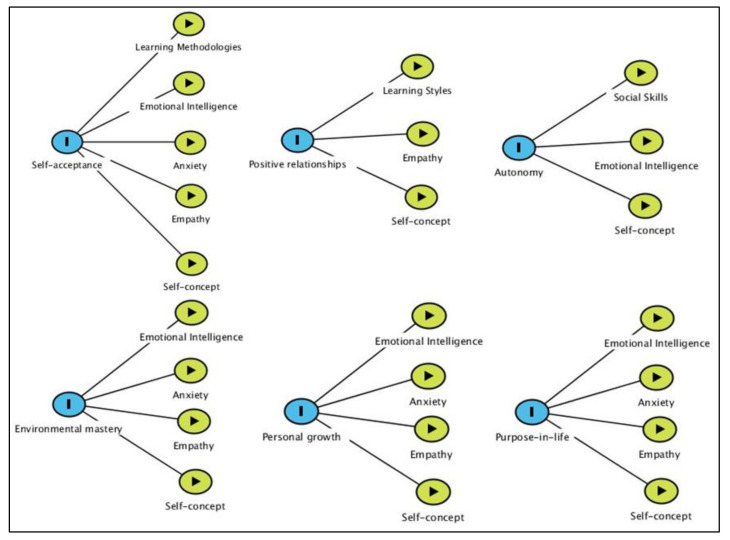
Directed acyclic graph of the relationship between the psychological well-being dimensions and psychosocial factors. Source: created with DAGitty.

Figure 1 depicts the relationship between the psychological well-being dimensions and psychosocial factors. Table 3 shows the final multiple regression models of the psychological well-being dimensions after the selection of independent variables.

The multivariate linear regression analysis involving the students on the Pedagogy program indicated that state anxiety, family self-concept, traditional learning methodologies and empathy (emotional understanding) were significantly related to environmental mastery, predicting 54% of its total variance (R^2^ = 0.540, F = 9.990, *p* < 0.001). For students on the Speech Therapy program, state anxiety and emotional intelligence (emotional attention) were significant predictors of the psychological well-being dimension (R^2^ = 0.593, F = 21.110, *p* < 0.001), accounting for 59.3% of its total variance. For student participants enrolled on the Pedagogy course, the results demonstrated that state anxiety, social responsibility and theoretic learning style were significantly related to purpose-in-life, predicting 49% of its total variance (R^2^ = 0.492, F = 11.306, *p* < 0.001). In the Speech Therapy program, the perspective taking, empathic stress and empathic happiness empathy dimensions predicted 57.3% of the total variance of this dimension (R^2^ = 0.573, F = 12.528, *p* < 0.001). In Table 4, the independent multiple regression models for the psychological well-being dimensions of environmental mastery and purpose-in-life among the students of the Pedagogy and Speech Therapy courses are shown.

## 4. Discussion

This study investigated the possible association between self-reported levels of psychological well-being and educational aspects (learning style and methodology, social skills and level of social responsibility) and psychological or cognitive-affective skills (emotional intelligence, anxiety, empathy and levels of self-concept). The final regression models indicated that the psychological well-being dimension of self-acceptance appears to be related to the cooperative learning methodology, the clarity dimension of emotional intelligence, state anxiety, the emotional understanding dimension of empathy and physical and family self-concept. The positive relationships dimension appeared to be related to the active learning style, the emotional understanding and empathic stress dimensions of empathy, academic/professional self-concept, social self-concept and family self-concept. Autonomy was associated with the emotional attention and clarity dimensions of emotional intelligence, emotional self-concept and social skills. Environmental mastery was related to global emotional intelligence, state anxiety, the emotional understanding dimension of empathy and family self-concept. Personal growth was associated with the emotional attention dimension of emotional intelligence, state anxiety, the emotional understanding dimension of empathy and academic/professional self-concept. Finally, purpose-in-life was related to global emotional intelligence, state anxiety, the empathic happiness dimension of empathy and family self-concept.

### 4.1. Learning Styles

Consistent with the emotional skills model [5,23,34], the results of this study demonstrate the importance of educational and socio-emotional constructs on psychological well-being. Nevertheless, the current study focuses on a particular group of university students (Social Education, Pedagogy and Speech Therapy) that could be more sensitized than others to the study variables due to the nature of these disciplines in themselves. These disciplines usually give help and service to people with disabilities. Specifically, the present study demonstrates that Pedagogy and Speech Therapy students exhibited differences for the two psychological well-being dimensions of environmental mastery and purpose-in-life. Pedagogy students demonstrated that state anxiety, family self-concept, traditional learning methodologies and empathy (emotional understanding) were significantly related to environmental mastery, and that state anxiety, social responsibility and theoretic learning style predicted purpose-in-life. For students enrolled in the Speech Therapy program, state anxiety and emotional intelligence (emotional attention) predicted the environmental mastery dimension, and the empathy dimensions of perspective taking, empathic stress and empathic happiness predicted purpose-in-life.

Within the two learning methodology options evaluated, a direct and significant relationship has been found between the cooperative learning methodology and the psychological well-being dimensions of self-acceptance, environmental mastery, personal growth and purpose-in-life. This indicates that a more cooperative learning methodology is more positive for students than the traditional one, since their learning could be greater and improved in quality [65,66]. Therefore, students who prefer this methodology will have a higher probability of showing higher psychological well-being. However, the results have shown that there is no significant relationship between learning style and psychological well-being in the population studied. Generally, while there is previous scientific literature including this educational variable, as far as we know, no conclusive results have been obtained about student preferences for one style or another, since this preference seems to vary with time and experience [67,68]. Another study found that the most effective active methodologies according to students were work and group dynamics [69]. Online, the higher the level of satisfaction and emotional well-being, the better the academic performance of students seems to be [70].

### 4.2. Social Skills

Regarding social skills, a significant direct relationship with all psychological well-being dimensions has also been observed. Therefore, the higher the level of social skills in these students, the higher the level of general psychological well-being. These results are consistent with those reported by Recabarren [71], Vasilenko et al. [31], Souri and Hasanirad [72] and Freire et al. [73]. These authors concluded that a larger number of positive relationships in one’s own environment or context led to better coping strategies and more resilience or capacity to adapt. This strengthening of strategies is likely to produce higher levels of psychological well-being for students because they achieve a higher level of support and optimism. In relation to social responsibility, no significant association with psychological well-being has been found [72,73]. The reason for this could be that while social responsibility is based on positive values such as altruism, more individualistic and selfish behaviors may prevail in the current world. This construct could be affecting the psychological well-being of university students in Spain [33]. In some recent studies [74], social responsibility has been shown to be correlated directly with life satisfaction for students during university years. The higher the social skills, the higher the level of emotional intelligence [13,15], which is associated with better psychological well-being [23].

### 4.3. Emotional Intelligence

The emotional attention dimension was directly correlated with all psychological well-being dimensions and inversely correlated with the autonomy dimension. This finding may be interpreted as suggesting that the greater the emotional attention, the higher the level of psychological well-being. However, a high level of emotional attention seems to imply a regression in the student’s level of autonomy. In the case of the clarity dimension, a direct association with all psychological well-being dimensions was observed. The reparation dimension had a direct relationship with the psychological well-being dimensions of self-acceptance, positive relationships, autonomy and mastery of the environment. This may mean that students who have greater repair emotional intelligence achieve better self-acceptance and autonomy, along with better mastery of the environment, which in turn fosters more positive relationships. Overall, high emotional intelligence in the student sample group appeared to contribute to high psychological well-being. The results in the current study highlight the importance of emotional skills in relation to psychological well-being, being a type of psychosocial adaptation to the educational environment [75,76]. For example, authors such as Burris et al. [77] point out how good management of emotional intelligence influences psychological well-being. These authors found a relationship between optimism and high psychological well-being in the university stage and pointed out that, in order to achieve this, one must have a defined awareness of one’s own emotional intelligence in order to actively train optimism in this group [77].

### 4.4. Anxiety

If anxiety levels are considered, both of these (state and trait) showed a significant but inverse relationship with the psychological well-being dimensions. State anxiety shows a relationship with all the well-being dimensions, although the highest significant association was observed with the environmental mastery dimension. This can be interpreted as meaning that the greater the state of anxiety that students feel at the present moment, the lower their levels of general psychological well-being in all its dimensions. In contrast, trait anxiety, characterized by a more stable construct over time, was only inversely related to the positive relationships dimension. It follows that students who have the personality trait of anxiety will have greater difficulties in establishing positive relationships with their environment. It can be concluded that, in keeping with the studies of Cooke et al. [4], Recabarren [71] and Stallman [6], higher levels of anxiety occur in the university stage and psychological well-being declines worryingly. Zuñiga [51] pointed out that state anxiety and trait anxiety seem to be correlated with all psychological well-being dimensions, except the personal growth dimension, applying the same instruments to assess anxiety and well-being in university students.

### 4.5. Empathy

Within the empathy dimensions, the emotional understanding dimension has been shown to be directly related to all psychological well-being dimensions, although the highest significant correlation has been obtained with the personal growth dimension. Therefore, students who are most able to understand their emotions and the emotions of others seem to show higher levels of overall psychological well-being. These results are also similar to those found by Cicognani et al. [78], where they show the importance of a sense of community, participation and social understanding for psychological well-being. In line with this, Gustems Carnicer and Calderón [42] found that students at high risk of psychological distress were more likely to show empathic stress and avoidance coping strategies. As in our study, other authors such as Serrano and Andreu [43] and Cañero et al. [5] showed, in the Spanish university context, that empathy was a predictor of subjective well-being in university students. Likewise, Malhotra and Kaur [44] also found a relationship between emotional empathy and perceived well-being. Sánchez-López et al. [79] found no significant correlation between psychological well-being and empathy in a study they reported.

### 4.6. Self-Concept

The levels of self-concept in the academic/professional, social and physical areas have been found to have a direct relationship with all psychological well-being dimensions. Therefore, students who have a good concept of themselves, both physically and in their studies and social relationships, appear to have a higher level of psychological well-being. The family self-concept dimension has a direct relationship with all psychological well-being dimensions except for the autonomy dimension. This relationship may indicate that, although at a general level a high self-concept in the family environment leads to high psychological well-being, this construct does not generate greater or lesser feelings of autonomy. In this line, Kazarian [80] has pointed out that having good family relationships influences students’ personalities and entails an improvement in psychological well-being and in one’s concept of oneself. In another study by Sánchez-López et al. [79], it was reported that self-esteem constitutes one of the elements that determines the so-called emotional intelligence profile and it was determined that positive relationships existed between self-esteem and subjective psychological well-being and material psychological well-being. Likewise, Fernández-Zabala et al. [74] found direct correlations between some self-concept dimensions and the satisfaction with life variable in university students. Other recent research [63] has also found that self-concept was one of the predictors of life satisfaction. In turn, emotional self-concept has been found to have an inverse relationship with four of the psychological well-being dimensions: self-acceptance, autonomy, environmental mastery and personal growth. This result may imply that a high emotional self-concept may generate a negative impact on these psychological well-being dimensions. In this way, some studies report that the greater the sense of humor (which is more frequent in people with greater positive self-concept), the greater their level of psychological well-being [81].

### 4.7. Limitations

Participants took part in the study on a voluntary basis and were not randomly selected. This research only used self-reported measures (i.e., self-rating scales) to evaluate psychological well-being and the set of constructs from the psycho-educational approach which may have been subject to recall bias. As a result, respondent bias may have been present due to social desirability. Additionally, participants may have answered items on the social responsibility questionnaire and the TECA in a socially desirable manner. Hence, objective evaluations would be more appropriate to evaluate the constructs examined in this study. Students were recruited from a single geographical location, so the generalizability of the results may be limited. The nature of the courses taken by the students in the study sample (Degree in Social Education, Pedagogy and Speech Therapy) may have sensitized them (had an influence) to the study variables response, therefore impacting on the results. Likewise, all of the student participants were recruited from the first year of the three courses which may have impacted their answers on the self-report questionnaire. For this reason, the interpretation of the findings for other similar populations of university students should be approached with caution.

### 4.8. Future Research

Future studies should evaluate the impact of educational initiatives taking into account the psychological well-being of university students and their learning styles, social skills, emotional intelligence, anxiety, empathy and self-concept and the emotional dimension, whether positive (emotional intelligence) or negative (anxiety), in university students as a group. However, since the study was conducted at one university site, future research in this topic should be made multi-center by including student data from several educational institutions. Additionally, multi-level analysis could be performed by taking into consideration specific needs and demands by geographical location or sociodemographic characteristic. In future research, it is important to test the role of possible moderators other than age, sex or academic discipline. A longitudinal study should be designed to evaluate the change in the variables investigated in this research as participants get older.

### 4.9. Implications for Education

Some possible educational implications arise from the current findings which may help to improve the learning process and its quality for students. The results can be used to design initiatives that increase the general perception of psychological well-being, prevent stress in the educational setting and lead to better academic performance.

The data could be used to implement academic development and an effective approach to the educational process and provide counselling and support services with strategies that will enhance students’ management of emotions and their ability to cope with certain conflict situations.

## 5. Conclusions

Among the psychological and cognitive dimensions selected, emotional intelligence, empathy, social skills and self-concept were positively correlated with all psychological well-being dimensions, whereas for learning methodologies, only the cooperative one was positively correlated with most of the psychological well-being dimensions. In contrast, anxiety was inversely correlated with the perception of well-being in university students, negatively influencing it. Therefore, it is necessary to look at this issue in more depth, providing more comments and observations on these results.

This research has shown that psychological well-being is associated with different psychological and educational constructs, both intra- and inter-personal. Firstly, students who prefer a cooperative learning methodology have greater self-acceptance. This implies their recognition of their worth and having better psychological well-being in themselves. Secondly, students who show a high capacity for adaptation and social skills use them to grow personally in situations that require this, obtaining higher levels of psychological well-being. Thirdly, we highlight the importance of students’ emotional skills, since a type of psychosocial adaptation that shows high emotional intelligence leads to high psychological well-being. Fourthly, in the university stage, there seem to be high levels of anxiety, generating psychological discomfort in students. Fifthly, the emotional understanding, both internal and external, of students seems to propitiate a high psychological well-being that could help them to grow personally. Finally, the importance of self-concept in all its dimensions, except the emotional one, has a significant influence on the psychological well-being of students. Having a good self-concept will, on the whole, produce higher levels of psychological well-being. Therefore, the individuals’ psycho-educational resources should be integrated and studied as a whole in relation to psychological well-being.

## Figures and Tables

**Table 1:** Descriptive results (range, mean, SD and quartile scores) of psychological well-being, learning style and methodology, social skills, social responsibility, emotional intelligence, anxiety, empathy and levels of self-concept in university students.

Measurement Variables	Min	Max	*Mean*	*SD*	Q1	Q2	Q3
**Psychological Well-Being**							
Self-acceptance	−4.00	20.00	11.02	5.08	8.00	11.50	15.00
Positive relationships	−17.00	8.00	−1.11	5.80	−5.00	−1.00	4.00
Autonomy	−18.00	12.00	−2.13	6.02	−6.00	−3.00	2.00
Environmental mastery	0.00	22.00	11.56	4.44	9.00	12.00	14.00
Personal growth	−1.00	21.00	12.53	4.81	9.00	13.00	16.00
Purpose-in-life	2.00	29.00	19.61	4.92	16.00	20.00	23.00
**Learning Styles**							
Active	5.00	85.00	12.77	6.61	11.00	12.00	14.00
Reflexive	7.00	74.00	15.70	5.44	14.00	16.00	17.00
Theoretic	6.00	81.00	15.33	6.11	13.00	16.00	17.00
Pragmatic	6.00	62.00	13.76	4.64	12.00	14.00	15.00
**Learning Methodologies**							
Traditional	10.00	50.00	31.44	8.34	27.00	32.00	37.00
Cooperative	51.00	125.00	95.54	11.99	89.00	95.00	102.00
**Social Skills**	21.00	78.00	65.46	8.34	60.00	65.00	73.00
**Social Responsibility**	120.00	194.00	149.76	13.77	140.75	149.00	159.00
**Emotional Intelligence**							
Emotional attention	14.00	40.00	27.28	5.99	23.00	27.00	31.25
Clarity	14.00	38.00	25.54	4.98	22.00	25.00	29.00
Repair	16.00	45.00	31.69	4.78	28.50	31.00	35.00
**Anxiety**							
State Anxiety	−30.00	21.00	−9.82	10.25	−17.00	−11.00	−2.50
Trait Anxiety	−2.00	14.00	3.41	1.97	2.00	4.00	4.00
**Empathy**							
Perspective taking	0.00	103.00	13.61	8.90	10.00	13.00	17.00
Emotional understanding	−1.00	26.00	13.30	6.06	8.00	13.00	18.00
Empathic stress	−11.00	88.00	4.50	8.55	0.75	3.50	8.00
Empathic happiness	7.00	169.00	23.76	13.14	20.00	23.00	27.00
**Self-concept**							
Academic/Professional	23.00	594.00	422.49	109.57	372.50	445.00	493.50
Social	12.00	396.00	274.01	76.44	238.75	289.00	326.75
Emotional	25.00	532.00	264.62	121.74	169.25	263.50	339.25
Family	−74.00	238.00	127.09	73.72	75.00	158.00	187.00
Physical	21.00	565.00	324.15	124.02	232.75	330.00	425.00

SD = standard deviation; Q = quartile scores.

**Table 2:** Correlations between psychological well-being and age, sex, learning style and methodology, level of social responsibility, empathy, anxiety, social skills, level of self-concept and emotional intelligence in university students.

Psychological Well-Being Dimensions/Demographic and Psychosocial Variables	Self-Acceptance	Positive Relationships	Autonomy	Environmental Mastery	Personal Growth	Purpose-in-Life
**Age**	0.000	−0.052	0.041	−0.034	−0.015	−0.078
**Sex**	−0.029	0.093	−0.086	−0.006	0.103	0.039
**Learning Style**						
Active	0.102	−0.063	0.014	−0.085	-0.068	0.046
Reflexive	0.002	−0.141	-0.018	−0.145	-0.079	−0.026
Theoretic	0.026	−0.126	-0.005	−0.157	-0.091	−0.016
Pragmatic	0.028	−0.166	0.030	−0.150	-0.125	−0.003
**Learning Methodology**						
Traditional	−0.125	−0.026	−0.092	−0.064	−0.131	−0.119
Cooperative	0.362^**^	0.148	0.069	0.220^*^	0.265^**^	0.292^**^
**Social Skills**	0.242^**^	0.251^**^	0.288^**^	0.261^**^	0.410^**^	0.263^**^
**Social Responsibility**	0.044	−0.085	−0.012	−0.131	−0.060	0.006
**Emotional Intelligence**						
Emotional attention	0.218^**^	0.279^**^	−0.066	0.175^*^	0.230^**^	0.279^**^
Clarity	0.443^**^	0.262^**^	0.423^**^	0.486^**^	0.236^**^	0.350^**^
Repair	0.236^**^	0.151	0.170^*^	0.230^**^	0.097	0.163
**Anxiety**						
State Anxiety	−0.527^**^	−0.336^**^	−0.411^**^	−0.547^**^	−0.303^**^	−0.454^**^
Trait Anxiety	−0.029	−0.188^*^	−0.091	−0.120	−0.148	−0.095
**Empathy**						
Perspective taking	0.130	0.056	0.081	−0.027	0.158	0.126
Emotional understanding	0.298^**^	0.382^**^	0.331^**^	0.376^**^	0.448^**^	0.326^**^
Empathic stress	0.046	0.037	−0.102	−0.104	−0.011	0.041
Empathic happiness	0.123	0.037	0.036	−0.049	0.068	0.133
**Self-concept**						
Academic/professional	0.313^**^	0.237^**^	0.224^**^	0.300^**^	0.460^**^	0.326^**^
Social	0.382^**^	0.477^**^	0.276^**^	0.400^**^	0.423^**^	0.329^**^
Emotional	−0.212^*^	−0.151	−0.344^**^	−0.245^**^	−0.171^*^	−0.158
Family	0.451^**^	0.291^**^	0.145	0.368^**^	0.376^**^	0.386^**^
Physical	0.526^**^	0.260^**^	0.245^**^	0.374^**^	0.206^*^	0.399^**^

*p < 0.05; **p < 0.001.

**Table 3:** Influence of psychosocial factors on psychological well-being in university students.

Self-Acceptance (R^2^ = 0.586)						
Independent Variables	B	95% CI		*β*	*SE*	*p*-Value
		**Lower bound**	**Upper bound**			
Cooperative Learning (Learning Methodologies)	0.075	0.018	0.232	0.182	0.029	0.010
Clarity (Emotional Intelligence)	0.157	0.002	0.311	0.146	0.078	0.047
State Anxiety	−0.140	−0.209	−0.070	−0.277	0.035	<0.001
Emotional Understanding (Empathy)	0.144	0.020	0.268	0.163	0.063	0.023
Physical Self-concept	0.012	0.006	0.018	0.279	0.003	<0.001
Family Self-concept	0.019	0.009	0.029	0.265	0.005	<0.001
**Positive relationships (R^2^ = 0.520)**						
**Independent variables**	**B**	**95% CI**		***β***	***SE***	***p*-value**
		**Lower bound**	**Upper bound**			
Active Learning Style	−0.379	−0.666	−0.093	−0.195	0.144	0.010
Emotional Understanding (Empathy)	0.263	0.107	0.419	0.261	0.079	0.001
Empathic Stress (Empathy)	0.392	0.205	0.580	0.309	0.094	<0.001
Academic/Professional Self-concept	−0.022	−0.033	−0.012	−0.382	0.005	<0.001
Social Self-concept	0.049	0.034	0.065	0.596	0.008	<0.001
Family Self-concept	0.018	0.006	0.030	0.227	0.006	0.003
**Autonomy (R^2^ = 0.313)**						
**Independent variables**	**B**	**95% CI**		***β***	***SE***	***p*-value**
		**Lower bound**	**Upper bound**			
Social Skills	0.177	0.040	.0315	0.219	0.069	0.012
Emotional Attention (Emotional Intelligence)	0.246	−0.430	−0.061	−0.247	0.093	0.010
Clarity (Emotional Intelligence)	0.484	0.252	0.715	0.391	0.117	<0.001
Emotional Self-concept	−0.011	−0.020	−0.002	−0.217	0.004	0.014
**Environmental mastery (R^2^ = 0.489)**						
**Independent variables**	**B**	**95% CI**		***β***	***SE***	***p*-value**
		**Lower bound**	**Upper bound**			
Global Emotional Intelligence	0.114	0.054	0.174	0.291	0.030	<0.001
State Anxiety	−0.160	−0.221	−0.100	−0.397	0.030	<0.001
Emotional Understanding (Empathy)	0.132	0.054	0.174	0.186	0.054	0.016
Family Self-concept	0.013	0.005	0.022	0.238	0.004	0.002
**Personal growth (R^2^ = 0.354)**						
**Independent variables**	**B**	**95% CI**		***β***	***SE***	***p*-value**
		**Lower bound**	**Upper bound**			
Emotional Attention (Emotional Intelligence)	0.131	0.003	0.260	0.164	0.065	0.046
State Anxiety	−0.143	−0.218	−0.069	−0.306	0.038	<0.001
Emotional Understanding (Empathy)	0.214	0.078	0.349	0.260	0.068	0.002
Academic/Professional Self-concept	0.015	0.007	0.023	0.319	0.004	<0.001
**Purpose-in-life (R^2^ = 0.439)**						
**Independent variables**	**B**	**95% CI**		***β***	***SE***	***p*-value**
		**Lower bound**	**Upper bound**			
Global Emotional Intelligence	0.119	0.043	0.194	0.252	0.038	0.002
State Anxiety	−0.135	−0.212	−0.058	−0.278	0.039	0.001
Empathic Happiness (Empathy)	0.274	0.094	0.454	0.249	0.091	0.003
Family Self-concept	0.017	0.006	0.027	0.245	0.005	0.003

*R^2^*, regression coefficient of determination; B, regression coefficient; *CI*, confidence interval; *β*, adjusted coefficient from multiple linear regression analysis; *SE* coefficient standard error.

**Table 4:** Influence of psychosocial factors on psychological well-being in university students according to the academic program—Pedagogy or Speech Therapy.

Environmental Mastery (R^2^ = 0.540) for Pedagogy						
Independent Variables	B	95% CI		*β*	*SE*	*p*-Value
		**Lower bound**	**Upper bound**			
State Anxiety	−0.177	−0.276	−0.078	−0.429	0.049	0.001
Family Self-concept	0.015	0.001	0.029	0.272	0.007	0.034
Traditional Learning (Methodology)	0.189	0.072	0.307	0.423	0.058	0.002
Emotional Understanding (Empathy)	0.257	0.053	0.461	0.339	0.100	0.015
**Environmental mastery (R^2^ = 0.593) for Speech Therapy**						
**Independent variables**	**B**	**95% CI**		***β***	***SE***	***p*-value**
		**Lower bound**	**Upper bound**			
State Anxiety	−0.225	−0.312	−0.138	−0.638	0.042	0.000
Emotional Attention (Emotional Intelligence)	0.205	0.053	0.358	0.331	0.075	0.010
**Purpose-in-life (R^2^ = 0.492) for Pedagogy**						
**Independent variables**	**B**	**95% CI**		***β***	***SE***	***p*-value**
		**Lower bound**	**Upper bound**			
State Anxiety	−0.253	−0.365	−0.140	−0.553	0.055	0.000
Social Responsibility	0.153	0.070	0.237	0.479	0.041	0.001
Theoretic Learning Style	−0.752	−1.273	−0.231	−0.379	0.257	0.006
**Purpose-in-life (R^2^ = 0.573) for Speech Therapy**						
**Independent variables**	**B**	**95% CI**		***β***	***SE***	***p*-value**
		**Lower bound**	**Upper bound**			
Empathic happiness (Empathy)	0.744	0.143	1.075	0.689	0.162	0.000
Empathic Stress (Empathy)	0.413	0.066	0.760	0.320	0.170	0.021
Perspective taking (Empathy)	−0.349	−0.688	−0.010	−0.302	0.166	0.044

*R^2^*, regression coefficient of determination; B, regression coefficient; *CI*, confidence interval; *β*, adjusted coefficient from multiple linear regression analysis; *SE* coefficient standard error.
